# Driving to Childhood Cancer Hub Hospitals: A Study on Hospital Accessibility in Japan

**DOI:** 10.31557/APJCP.2020.21.6.1725

**Published:** 2020-06

**Authors:** Anna Tsutsui, Yukari Taniyama, Yuko Ohno

**Affiliations:** *Department of Mathematical Health Science, Graduate School of Medicine, Osaka University, Suita, Japan.*

**Keywords:** Hospitals, pediatric, child, adolescent, neoplasms, health services accessibility

## Abstract

**Objective:**

In 2013, 15 childhood cancer hub hospitals in Japan were designated to provide quality medical treatment and care. The present study assessed hospital accessibility by investigating travel times and distances from patient residences.

**Methods:**

A total of 37,309 residence/hospital pairs were generated using the addresses of 15 hub hospitals that were designated in 2019 and local government offices in 2014. Using the Google Directions Application Programming Interface (API), travel times and distances were calculated on the assumption that each patient would arrive by driving to the hospitals by 10 am on Wednesday, November 6, 2019. Thus, after identifying the nearest hospital for each residence and deriving adjusted estimated travel times (AETT), the data were summarized according to the regional block using weighted population descriptive statistics for children under 15 years of age in 2015. The cumulative distribution functions of the weighted mean of AETT were also plotted.

**Results:**

Childhood cancer patients could access the nearest hub hospital by traveling approximately 1.78 hours (AETT, range: 0.1 to 41.8) and 91.86 km (range: 1.0 to 1438.0). Moreover, a total of 94.5% of patients had the nearest hub hospital within their own regional block. The cumulative distribution functions of AETT indicated that many children in three blocks with multiple hub hospitals have shorter travel times and better hospital accessibility than those in other blocks.

**Conclusions:**

Although feasibility is ultimately dependent on each patient’s condition and situation, child cancer patients on average can likely complete hospital visits from home and return within a single day. However, this is likely not the case for children who live at considerable distances from hub hospitals. We found regional differences in travel times and distances, depending on whether a given block contained multiple hub hospitals.

## Introduction

It is rare for cancer to affect children between 0 and 14 years of age. Nonetheless, the disease affects many children throughout the world. In 2015, estimates indicated that there were 397,000 incidences worldwide (Ward et al., 2019). In Japan alone, yearly incidence was estimated at 2,055, with a crude incidence rate of 122.7 per million persons between 2009–2011 (Katanoda et al., 2017). The Japanese universal health insurance system provides free hospital access to these and other patients. As a result, there was concern that many hospitals were treating only a handful of affected children each year. Indeed, hospital case volumes for childhood cancer treatment were lower than those observed in the United Kingdom or Georgia (Tsutsui et al., 2009). However, the centralization of cancer hospitals was an important measure for improving survival rates (Knops et al., 2013). One study also reported the need to increase psychological care for childhood cancer patients and their families while also ensuring that these individuals had adequate accommodations during therapy (Sakaguchi et al., 2014). Furthermore, childhood cancer survivors have higher risks of incurring a broad range of health conditions resulting from the long-term negative effects associated with some medical treatments and/or cancer types (Armstrong et al., 2014; Ishida et al., 2016; Furui et al., 2019). In sum, the centralization of these treatment hospitals is an important cancer control measure that will eventually ensure quality medical treatment and care.

As for cancer control measures in Japan, the Japanese Cancer Control Act was enforced in 2007 (Moore and Sobue, 2009). National and local governments then established the Basic Plan to Promote Cancer Control Programs. The issue of childhood cancer was included for the first time when the plan was later revised in 2012. To increase centralization and cooperation between all hospitals in Japan (Nakata et al., 2018), all requirements for childhood cancer hub hospitals were revealed in 2012. Thus, a total of 15 hospitals – across seven regional blocks – were designated as hub hospitals in 2013. In 2018, the Japanese government also issued revised guidance to update this system to promote collaboration with other local hospitals. Afterward, a few hub hospitals were replaced in 2019.

**Figure 1:**
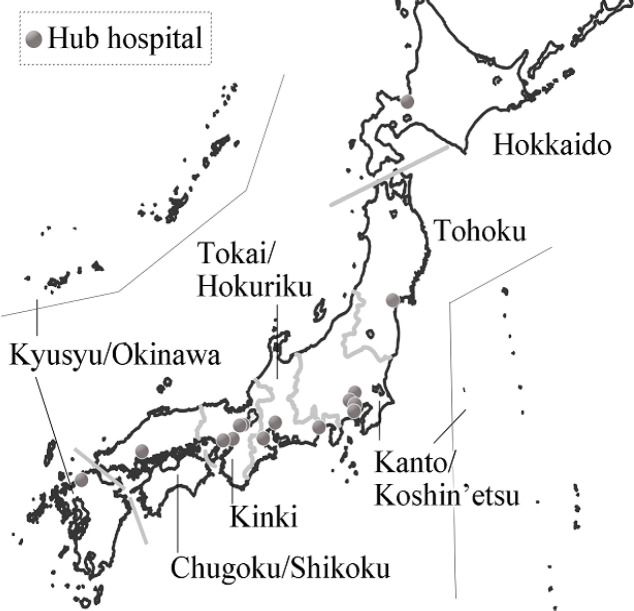
Seven Regional Blocks and Childhood Cancer Hub Hospitals in Japan

Figure 1 shows the latest 15 hub hospitals as of April 1, 2019 (Ministry of Health, Labour and Welfare, 2019). Currently, there are one to four hub hospitals per regional block. As a result, travel times may be much longer in areas located at a distance from these hub hospitals. These travel times are therefore highly likely to affect hospital selections for the parents of children with cancer. In fact, many parents have expressed concerns over future travel times. Sakaguchi et al., (2014) reported that more than 96% of parents whose children had received cancer treatment responded that travel times lasting more than 2 hours one-way for hospitalizations and outpatient clinic visits were unacceptable. However, no previous Japanese studies have evaluated healthcare access—from the perspective of travel times and distances—from patient residences to childhood cancer hub hospitals (Tanaka et al., 2018). Therefore, the present study investigates travel times and distances to evaluate the burdens placed on these children and their families in Japan.

## Materials and Methods

This was a simulation study designed to investigate travel times and distances from patient residences to hub hospitals. Thus, we retrieved existing data and generated residence/hospital address pairs. Travel times and distances by driving for each pair were then acquired through the Google Directions Application Programming Interface (API). These results were then summarized and plotted.

*Data sources*

To investigate travel times and distances, we required information on destinations and origins. In this context, destinations were defined as the addresses of the 15 aforementioned childhood cancer hub hospitals, as of April 1, 2019. This information was available on the web (Ministry of Health, Labour and Welfare, 2019). Origins were defined as the residences of child patients from all over Japan. Given that such information was unavailable, we alternatively used the addresses of the local government offices, including branch and contact offices in 2014 (n=5,774) (National Land Numerical Information Download Service, 2014). We also assumed that travel time and/or distance values in regions with large populations would have greater impacts on our analysis. Thus, the childhood population under 15 years of age from the 2015 census was included to calculate weighted means (Portal Site of Official Statistics of Japan, 2019). In sum, we retrieved data on all destinations, origins, and area populations from public internet sources. These data were then used for analysis.

A total of 31 local government offices related to Fukushima prefecture were excluded from analysis (n=5,743). As a result of damage incurred from the Great East Japan Earthquake and nuclear disaster of 2011, specific locations in Fukushima prefecture were designated as restricted areas by the Japanese government (Nuclear Emergency Response Headquarters, 2011), causing residents and local government offices to move to outside locations. Thus, for the purposes of our analysis, 24 offices were excluded because they were still located outside their original areas as at 2014, while seven others were excluded because population data were not available during the 2015 census. 

*Residence/hospital pairs*

Using the cartesian product method, we generated 86,145 residence/hospital address pairs from each residence (n=5,743) to each hospital (n=15). The cartesian product of R and H (denoted as R×H) is the set defined as follows:

In this definition, R is a set of residence addresses, H is a set of hospital addresses, and (r, h) denotes an ordered pair (Bloch, 2011). Thus, our collection of pairs included all possible residence-to-hospital address combinations throughout Japan. That is, the collection certainly included many pairs that were not geographically nearest to one another. The following pairs were therefore extracted from the resulting collection: (1) 13,732 with addresses located in the same regional block and (2) 23,577 with addresses located in different but adjacent regional blocks. Thus, a total of 37,309 pairs were used for the API request.

*Google Directions API*

Many previous studies have evaluated healthcare access by estimating travel times and/or distances using Geographic Information Systems (GIS) software. However, GIS software requires the input of a well-defined road network dataset and significant effort in implementing the task; additionally, the skillful usage of related software presents a major obstacle (Wang and Xu, 2011). Therefore, many researchers are now adopting a web service, called web API, as a new and simple method to conduct similar analyses (Shaw et al., 2017; Sommerhalter et al., 2017). Of particular note is the Google Maps Platform service and its various web APIs (Google Maps APIs). This service offers nearly the same functions as the Google Maps website for obtaining directions (http://maps.google.com). It enables any software, including SAS, R, and Python, to send requests for directions to the API by a Hypertext Transfer Protocol (HTTP) request and then to receive the response directly. Google Maps APIs have several advantages: there is no need to prepare a road network and they offer more updated road networks compared with the commonly used GIS, ArcGIS Network Analyst module (Wang and Xu, 2011). Thus, we adopted the Google Directions API, one of Google Maps API, to obtain travel times and distances.

Google Directions API provides directions for several travel modes, including “Driving” and “Transit”, according to the user’s request; however, the “Transit” mode that uses the public transit system was unavailable in Japan at the time of our study (Google Maps Platform, 2019). Therefore, we adopted the “Driving” mode. In this mode, we assumed that drivers would not use any public transit systems or airplanes, but they could use cars or ferries. That is, residents living on the island were expected to always use ferries instead of airplanes; as such, travel times naturally increased. The directions in the “Driving” mode also considered travel time as the primary factor, while other factors included overall distance, number of turns, road network, and average time-independent traffic conditions if no departure time was specified (Google Maps Platform, 2020). Once a request was sent by a user, the API sent a response as the result of the abovementioned calculations, including estimated travel distances (ETD) in meters, estimated travel times (ETT) in seconds, and detailed directions.

Finally, we adopted the following settings for sending requests to the API: The travel mode was set to “Driving” for an arrival date/time of 10 am on Wednesday, November 6, 2019. This specific time was selected because it was one hour before 11 am, which we determined was the earliest reception closure time at all 15 investigated hub hospitals. All requests were sent on October 8, 2019.

*Statistical analysis*

Statistical analysis was conducted on the data acquired from the API as outlined above. Given that multiple results were acquired for each residence (i.e., multiple residence/hospital pairs), the nearest respective hospitals were first identified by referring to minimum ETTs. 

The primary outcome measure was the mean of adjusted estimated travel times (AETT) in hours. AETT was derived by totaling ETT and rest times, assuming that drivers would take one 15-minute break every two hours. We thought that all travel times should include these breaks because the API request was set to “Driving” and many ETTs acquired from the API were considered long-distance travels.

The other outcome measures were set as the means of ETD, proportions of children whose nearest hub hospital was in their regional block, and children who needed to travel via ferry. Estimated departure times (in clock hours) were also investigated. These times were derived by subtracting each AETT from 10 am as the arrival time.

The means of each outcome measure were calculated according to the city/ward/town/village of each prefecture. Using these values, we then calculated weighted population descriptive statistics for children under 15 years of age in 2015 according to seven regional blocks. This analysis was also performed after excluding residences that required ferry travel. 

Cumulative distribution functions were plotted using the weighted means of AETT. Finally, a choropleth map was created using estimated departure times. SAS 9.4M5 was used to acquire API data and to conduct the statistical analyses.

## Results

Of the 37,309 total API requests, there were 36,877 valid results. For the 432 that failed, residences were on the island, but ferry schedules were likely unavailable in the Google Maps service. The nearest hospitals were identified for 5,674 residences from all valid results. 

Table 1 shows a summary of travel characteristics and approximate travel times and distances to hub hospitals by regional block. In all regional blocks, average AETT was 1.78 hours (range: 0.1 to 41.8), and EDT was 91.86 km (range: 1.0 to 1438.0). This indicates that residents moved an average of 51.6 km per hour. Maximum AETT was 41.8 hours in the Kyushu/Okinawa block, which still represented a high value of 7.3 hours in the Hokkaido block, even when residences that required ferry service were excluded. Average AETT ranged from 0.78 to 5.73 hours between blocks, with a 7.3-fold difference in average AETT. These differences decreased when residences that required ferry service were excluded (0.78 to 2.18 hours and 2.8-fold). Below average AETT values were found in Kinki, Tokai/Hokuriku, and Kanto/Koshin’etsu blocks. Furthermore, 94.5% of all residences had their nearest hospitals within the same block. Hokkaido and Kyushu/Okinawa blocks had the highest values (100%), with the lowest found in the Chugoku/Shikoku block (63.5%). While only 2.1% of residences required ferry use, the percentage remarkably increased to 14.5% in the Kyushu/Okinawa block.

**Figure 2:**
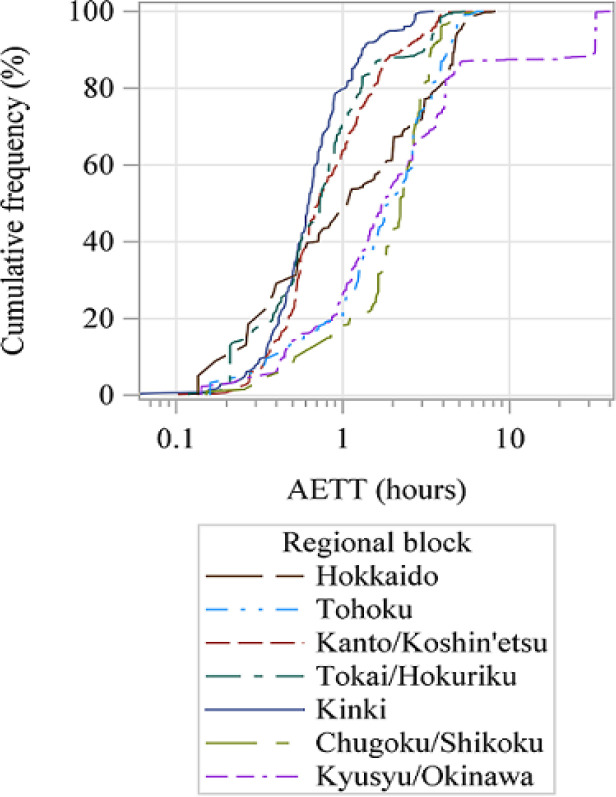
Cumulative Distribution Functions of AETT by Regional Block

Figure 2 shows the cumulative distribution functions of AETT. There were three types of curves: those in Kinki, Tokai/Hokuriku, and Kanto/Koshin’etsu blocks increased sharply (i.e., at least 60% cumulative of all children were estimated to reach hub hospitals within one hour); those in Chugoku/Shikoku, Tohoku, and Kyushu/Okinawa blocks increased more gradually (i.e., 18.1 to 26.2% of all children were estimated to reach hospitals within one hour); and those in Hokkaido increased sharply at first, but then shifted to a more gradual curve (i.e., 48.2% of all children were estimated to reach hub hospitals within one hour).

**Figure 3:**
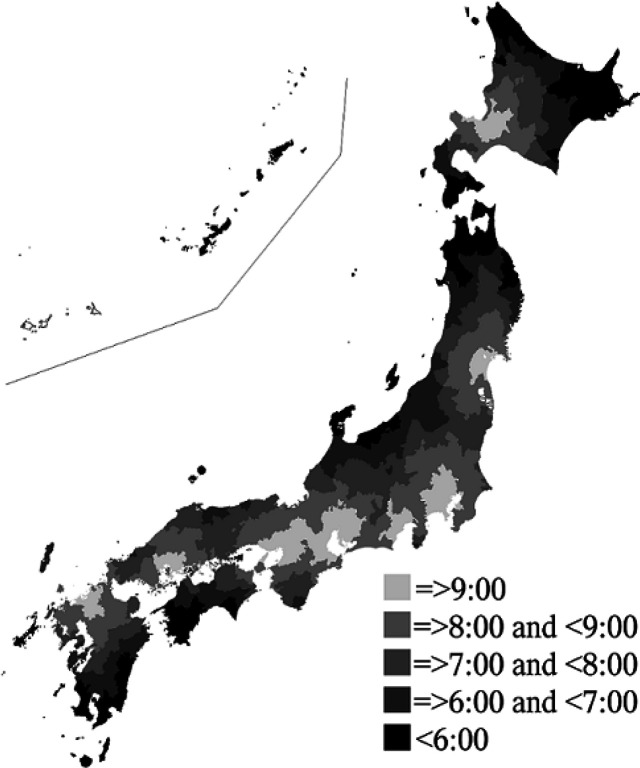
Choropleth Map of Estimated Departure Times to Hub Hospitals in Japan. White area indicates no available results. The Izu Islands and Ogasawara Islands in the Kanto/Koshin'etsu block were not plotted due to unavailable results

Figure 3 shows a choropleth map for estimated departure times. The farther the residence was from the hub hospital, the faster the departure time was concentrically. Departure times in the three regional blocks in the center of Japan (i.e., Kinki, Tokai/Hokuriku, and Kanto/Koshin’etsu) were later than those in other blocks. However, except for Kinki, all blocks contained residences requiring departures prior to 6 am. Many islands that required ferry service had earlier departure times than in nearby areas and some estimated departure times were earlier than those in nearby areas.

**Table 1:** Travel Characteristics and Approximate Travel Times and Distances to Hub Hospitals by Regional Block

Regional block	N of HPs†	Number of children per 100,000 persons (%)			Mean (Min, Max)		Mean (Min, Max) where ferries were not required†††	
		n††	Children who had Nearest HP within the same block	Children where ferries were required	AETT (h)	ETD (km)	AETT (h)	ETD (km)
All regional blocks	15	161.5	152.6 (94.5)	3.5 (2.1)	1.78 (0.1, 41.8)	91.86 (1.0, 1438.0)	1.30 (0.1, 7.3)	77.29 (1.0, 457.7)
Hokkaido	1	6.1	6.1 (100.0)	0.1 (1.5)	1.86 (0.1, 8.2)	118.13 (1.9, 449.9)	1.84 (0.1, 7.3)	117.62 (1.9, 449.9)
Tohoku	1	10.6	10.5 (99.8)	0.0 (0.2)	2.18 (0.2, 7.0)	149.84 (4.2, 457.7)	2.18 (0.2, 6.8)	149.73 (4.2, 457.7)
Kanto/Koshin'etsu	4	58.4	56.2 (96.1)	0.1 (0.1)	1.07 (0.1, 7.8)	58.98 (1.6, 358.0)	1.06 (0.1, 4.9)	58.68 (1.6, 329.3)
Tokai/Hokuriku	3	25.6	25.6 (99.9)	0	1.04 (0.1, 5.4)	59.86 (2.0, 382.4)	1.04 (0.1, 5.4)	59.86 (2.0, 382.4)
Kinki	4	27.1	25.6 (94.7)	0	0.78 (0.1, 3.5)	39.29 (1.0, 207.0)	0.78 (0.1, 3.5)	39.29 (1.0, 207.0)
Chugoku/Shikoku	1	14.2	9.0 (63.5)	0.4 (3.1)	2.20 (0.2, 7.5)	133.41 (2.6, 391.6)	2.17 (0.2, 6.3)	134.45 (2.5, 391.6)
Kyusyu/Okinawa	1	19.6	19.6 (100.0)	2.8 (14.5)	5.73 (0.1, 41.8)	235.11 (2.3, 1438.0)	1.89 (0.1, 5.5)	121.38 (2.3, 363.2)

N, Number; HP, Childhood cancer hub hospital; †† n, Number of child population per 100,000 persons; †††, This analysis excluded residences that required ferry travel.

## Discussion

To the best of our knowledge, this is the first study to investigate driving travel times and distances in regard to childhood cancer hub hospitals in Japan. By using the Google Maps API, travel times and distances were calculated in consideration of various factors (Google Maps Platform, 2020). In addition, previous studies have used the Google Distance Matrix API, a Google Maps API, to return ETT and ETD results only (Shaw et al., 2017; Sommerhalter et al., 2017). However, our study used the Google Directions API, which provided additional details for directions, in order to confirm whether ferry use was required and thus enabled us to exclude those areas from analysis when needed.

The centralization of childhood cancer treatment is important for improving the survival ratio (Knops et al., 2013), which has been a key concern in Japan. Even after the implementation of these hub hospitals, research indicates that childhood cancer hub hospitals only treated 33.6% of newly treated childhood cancer patients in 2016-2017 (Matsumoto et al., 2019). Thus, to promote centralization, it is necessary to evaluate hospital accessibility to hub hospitals.

As shown in Table 1, we found that patients needed to travel an average of approximately 1.78 hours and 91.86 km. Furthermore, almost all residents had their nearest hospitals within their respective blocks. Although feasibility is ultimately dependent on each patient’s condition and situation, child cancer patients on average are likely able to complete hospital visits from home and return within a single day, indicating that the hub hospitals were arranged to further promote the centralization of cancer treatment. However, previous research indicates that travel times greater than 2 hours are unacceptable for most parents in Japan (Sakaguchi et al., 2014). Hence, these long travel times may adversely affect the promotion of hospital centralization. 

Focusing on maximum values, some children required 3.5 hours or more of travel time across regional blocks. As far as we are aware, almost half of the hub hospitals that we examined closed their reception areas for first-visit patients at 11 am. Thus, we viewed patients with long travel times as being unable to complete hospital visits and to return home within a single day. Research indicates that childhood cancer patients and their families experience many burdens throughout their treatment (Eiser et al., 2006); thus, it is likely that long-distance travel may increase these burdens. Therefore, measures such as extending reception hours and shortening wait times in hospitals should decrease the overall time spent and thereby reduce related burdens. Furthermore, hospitalizations may last weeks or months in certain cases; thus, special hotels at nearby hospitals should be specifically provided to children and families who must travel far from home.

Differences between mean AETT were shown to compare blocks; lower values were found for three blocks with multiple hub hospitals, including Kinki, Tokai/Hokuriku, and Kanto/Koshin’etsu. As shown in Figure 2, the cumulative distribution functions of AETT indicate that many children in these blocks have shorter travel times and better hospital accessibility than those in other blocks. A similar trend was observed for estimated departure times (Figure 3). 

Figure 3 shows that children who live further away from hospitals need to depart from their homes earlier. There were also areas that required earlier departure times than those in neighboring locations. One reason for this is that government offices are located far from expressways; thus, times may be improved by extending expressways. As Tohoku block was particularly affected by the Great East Japan Earthquake of 2011, local roads/support roads are being reconstructed and are intended to reach completion by the fiscal year ending March 31, 2020 (Ministry of Land, Infrastructure, Transport and Tourism, 2017). Hence, travel times may be improved once this reconstruction is finished. 

Our results should be interpreted cautiously when viewing areas in the Kyusyu/Okinawa block. Here, nearest hospitals for all children were within the same block, and 14.5% of children required ferries to make their hospital visits. This block contains many islands and has direct flights to major airports around Japan. Therefore, these residents may have a greater need to travel by air rather than ferry, and thus may have the opportunity to select various hub hospitals outside their blocks. Given this, actual AETTs, ETDs, and percentages of residents who visited hub hospitals in their own blocks may differ from our results. However, even if using air-travel, the majority of residents will need to consistently depart one day earlier to arrive at a hospital by 10 am. These same cautions should be considered for other regional blocks. That is, with airfields all throughout Japan, airplanes are an option for residents who live far from hub hospitals or for children with cancers that are difficult to treat. Therefore, further studies are needed to consider air-travel options.

As for the API settings, we selected driving as the method of transportation due to current API restrictions (Google Maps Platform, 2019). Despite this issue, we considered this setting reasonable because children must be accompanied by parents/guardians when visiting hospitals. Equally as important, children with cancer are prone to infection; for example, in the case of influenza, children with cancer can develop severe complications (Goossen et al., 2013). In addition, children with cancer are usually advised by physicians and/or nurses to stay away from crowds and people who are sick (National Institutes of Health, 2015), thus they are expected to travel via car when available. 

Our study has several limitations. First, residential addresses were substituted for local government offices in Japan. Second, we did not consider specific ages, pathologies, or treatment needs of patients. Thus, further analyses based on registry data are needed to help evaluate current and ideal hospital arrangements. Third, in our results which we obtained from using the API, we did not consider a time-dependent traffic condition. This is important because real-world travel time may generate higher values compared to our results, due to traffic congestions during the morning commute. Fourth, according to Google, results may vary over time due to changes in road networks, updated average traffic conditions, and the distributed nature of the service (Google Maps Platform, 2020). To the best of our knowledge, Google has not released details regarding map data and algorithm calculations to the public. Thus, it is difficult to ensure the consistency of our results and to evaluate the accuracy of the search results. 

In conclusion, although feasibility is ultimately dependent on each patient’s condition and situation, we consider that child cancer patients, on average, are able to attend hospital visits and to return home within a single day. However, this is not the case for children who live considerable distances from hub hospitals. We uncovered regional differences in travel times and distances, depending on whether a given block contained multiple hub hospitals. Our study suggests that the estimated travel times and distances obtained from web services, including web APIs, can be beneficial not only for current private use but also for public use by researchers, healthcare providers, and healthcare policymakers. Therefore, further research from this perspective is needed to provide better-quality medical treatment.
